# *Zea mays* cultivation, biochar, and arbuscular mycorrhizal fungal inoculation influenced lead immobilization

**DOI:** 10.1128/spectrum.03427-23

**Published:** 2024-02-23

**Authors:** Qiong Jia, Jiahua Sun, Qiuyu Gan, Nan-Nan Shi, Shenglei Fu

**Affiliations:** 1 College of Geography and Environmental Science, Henan University, Kaifeng, China; 2 Key Laboratory of Geospatial Technology for the Middle and Lower Yellow River Regions, Henan University, Ministry of Education, Kaifeng, China; 3 Miami College of Henan University, Kaifeng, China; Huazhong Agricultural University, Wuhan, China

**Keywords:** AMF, biochar addition, plant cultivation, Pb fractions, Pb bioavailability, Pb phytoremediation

## Abstract

Plant cultivation can influence the immobilization of heavy metals in soil. However, the roles of soil amendments and microorganisms in crop-based phytoremediation require further exploration. In this study, we evaluated the impact of *Zea mays* L. cultivation, biochar application, and arbuscular mycorrhizal fungi (AMF) inoculation on soil lead (Pb) immobilization. Our results indicated that biochar addition resulted in a significant, 42.00%, reduction in AMF colonization. Plant cultivation, AMF inoculation, and biochar addition all contributed to enhanced Pb immobilization, as evidenced by decreased levels of diethylenetriaminepentaacetic acid- and CaCl_2_-extractable Pb in the soil. Furthermore, soil subjected to plant cultivation with AMF and biochar displayed reduced concentrations of bioavailable Pb. Biochar addition altered the distribution of Pb fractions in the soil, transforming the acid-soluble form into the relatively inert reducible and oxidizable forms. Additionally, biochar, AMF, and their combined use promoted maize growth parameters, including height, stem diameter, shoot and root biomass, and phosphorus uptake, while simultaneously reducing the shoot Pb concentration. These findings suggest a synergistic effect in Pb phytostabilization. In summary, despite the adverse impact of biochar on mycorrhizal growth, cultivating maize with the concurrent use of biochar and AMF emerges as a recommended and effective strategy for Pb phytoremediation.

**IMPORTANCE:**

Heavy metal contamination in soil is a pressing environmental issue, and phytoremediation has emerged as a sustainable approach for mitigating this problem. This study sheds light on the potential of maize cultivation, biochar application, and arbuscular mycorrhizal fungi (AMF) inoculation to enhance the immobilization of Pb in contaminated soil. The findings demonstrate that the combined use of biochar and AMF during maize cultivation can significantly improve Pb immobilization and simultaneously enhance maize growth, offering a promising strategy for sustainable and effective Pb phytoremediation practices. This research contributes valuable insights into the field of phytoremediation and its potential to address heavy metal pollution in agricultural soils.

## INTRODUCTION

In recent decades, global reliance on agriculture has grown, yet soil quality has significantly deteriorated, largely attributed to environmental pollution (1). Soil pollution induced by heavy metals (HMs) has emerged as a critical environmental concern due to its potential threat to ecosystems (2). Lead (Pb) is one of the most extensively distributed metal contaminants globally (3). Numerous human activities and industrial processes, including coal combustion, leaded gasoline manufacturing, mining, smelting, and electroplating, have contributed to the escalation of Pb pollution (4). Excessive Pb in agricultural soil can substantially impair soil quality and crop growth, leading to its accumulation in grains (5). Consequently, its subsequent entry into the food chain can give rise to chronic health problems in humans, including cardiovascular, hematological, and renal-related diseases (6). Effective remediation methods are required for Pb-contaminated soils to safeguard public health and food security.

Numerous physical and chemical methods have been developed to mitigate the risks of Pb pollution (7). Nonetheless, these conventional methods are often expensive and have the potential to cause irreparable harm to the soil environment (8). In contrast, phytoremediation has received a lot of attention recently due to its benefits, such as economic effectiveness, environmental friendliness, and *in situ* nature (9). Phytoremediation, a pivotal component of environmental remediation, can mitigate the bioavailability of HMs while concurrently improving soil quality through the utilization of metal-tolerant plants and additives (10). In cases of severe metal pollution, phytostabilization may represent the most cost-effective approach for remediating polluted soils (11). The cultivation of plants can decrease the mobility of Pb by binding it to bioactive molecules (12). Yet, the slow growth rates and limited biomass production of metal-tolerant plants can impose practical constraints on phytostabilization.

Biochar has recently garnered significant attention due to its potential to enhance the remediation of HMs in an economically viable and environmentally friendly manner (13). Biochar is a carbon-rich material produced via the thermal decomposition of biomass under the condition of a limited oxygen supply (14). When mixed into cropland soil, biochar can act as a fertilizer to promote soil fertility and plant growth by supplying and retaining nutrients in agricultural ecosystems (15). Biochar has also been found to stabilize and reduce the bioavailability of hazardous metals due to its alkaline and porous structure and cation exchange capacity (16). In accordance with meta-analytical research, biochar demonstrated the ability to reduce soil-extractable Pb by 47.1% and decrease Pb accumulation in plants by 39.1% (17). Consequently, biochar has been widely applied to promote the remediation of heavy metal-contaminated soils.

Biofertilizers such as arbuscular mycorrhizal fungi (AMF) are known for aiding in the phytostabilization of soils contaminated with heavy metals (18, 19). AMF are prevalent soil microorganisms that naturally associate with most terrestrial plants in both natural and agricultural soils (20, 21). Pb pollution has been documented to inhibit spore germination and root colonization by AMF due to the anti-fungal properties of HM compounds (22). Interestingly, AMF establishment is often observed in Pb-polluted mining sites (19). It has been suggested that host plants may regulate AMF colonization by providing more carbohydrates to AMF, as AMF offers protection against Pb toxicity (23). Moreover, the extraradical mycelium of AMF spread out and proliferates in the soil, forming extensive underground common mycelial networks. AMF can directly adsorb and fix Pb ions using mycelia or by secreting substances like glomalin-related soil protein, which enhances Pb retention in the soil and reduces its bioavailability to plants (24). Additionally, intracellular hyphae can modify phytotoxicity through enzymatic pathways to retain the metals (25). The utilization of AMF in conjunction with biochar is an appealing technology for phytoremediation due to its cost-effectiveness and synergistic benefits (26). Biochar addition may increase AMF abundance as a consequence of changes in soil resource reserves (carbon, nutrients, water, etc.) and soil characteristics [pH, cation exchangeable capacity (CEC), etc.] (27). Nevertheless, there is a lack of studies that comprehensively examine the combined effects of plant cultivation, AMF, and biochar on the distribution, mobility, and translocation of Pb fractions (28–30).

Maize, a high-quality food crop extensively cultivated globally, is readily colonized by AMF. Maize has been viewed as a promising plant for phytostabilization due to its rapid growth, high biomass, and resistance to HM in soils (31). However, the influence of plant cultivation, AMF colonization, and biochar application on soil Pb bioavailability, Pb uptake, and translocation remains uncharted. As far as we know, this is the first time to investigate the effect of maize cultivation, biochar application, and AMF inoculation on the distribution, mobility, and translocation of soil Pb. Our hypotheses are as follows: (i) plant cultivation, AMF inoculation, and biochar addition will reduce Pb bioavailability in soil; (ii) the application of AMF and biochar will synergistically promote maize growth while diminishing the transfer of Pb from soil to maize roots and from maize roots to shoots.

## MATERIALS AND METHODS

### Soil preparation

The surface soil (0–20 cm depth) utilized in this investigation was collected from a biological garden at Henan University, Henan, China. The soil properties were as follows: pH [8.3, 1:2.5 (wt/vol)], total organic carbon (13.6 g kg^−1^), total nitrogen (1.1 g kg^−1^), total phosphorus (0.5 g kg^−1^), available phosphorus (6.7 mg kg^−1^), and total Pb (14.95 mg kg^−1^). Subsequently, the soil was autoclaved for 2 hours at 121°C after passing through a 2 mm filter. Pb(NO_3_)_2_ was added to the soil and thoroughly mixed to achieve a concentration of 1,000 mg Pb per kg of dry soil. This Pb concentration was selected in accordance with the Soil Environmental Quality of China (GB 15168-2018). After Pb addition, the soil was maintained in darkness at 60% of its water-holding capacity for 1 month to stabilize.

### Plant material, biochar, and AMF inocula

Maize seeds (Zhengdan 958) were sterilized by soaking them for 5 minutes in 0.5% (vol/vol) NaClO, followed by multiple rinses with distilled water. These seeds were germinated on moistened filter paper in Petri dishes for 2 days.

Maize straw was used to produce biochar in this study. The original biomass was cut into 10 cm segments before biochar production. The muffle furnace employed for the pyrolysis process was heated under oxygen-limited conditions with a nitrogen flow. The temperature was gradually raised to 550°C at a rate of 10°C per minute and maintained at this temperature for 4 hours. Subsequently, the biochar was cooled, powdered, and sieved through a 2 mm mesh before application. Detailed biochar properties are presented in Table S1.

The AMF species, *Diversispora eburnea* (BGC HK02C, GenBank Accession No.: KT152858), was sourced from the Institute of Plant Nutrition and Resources at the Beijing Academy of Agriculture and Forestry. *D. eburnea* is an AMF species commonly found in the HM mine tailings and has been reported to have an effect on soil HM remediation (32). To propagate AMF, it was cultivated on *Zea mays* in autoclaved sandy soil for 6 months. The AMF inoculum comprised mycelium, spores (50 spores g^−1^ inoculum), maize root fragments (with an approximate 80% colonization rate), and soil.

### Pot trial

The experiment was conducted in a greenhouse using a completely randomized design. There were six treatment combinations: (i) control soil without plant cultivation, biochar, or AMF (CK); (ii) plant cultivation with maize (P); (iii) unplanted soil added with biochar (B); (iv) plant cultivation added with biochar (PB); (v) plant cultivation inoculated with AMF (PA); and (vi) plant cultivation added with biochar and AMF (PBA). Each treatment had five replicates, resulting in a total of 30 pots.

The pots, with a diameter of 13.5 cm and a height of 30 cm, were filled with 2 kg of soil prepared as described earlier. For AMF treatments (PA and PBA), 50 g of live inoculum was applied, while autoclaved inoculum (50 g) was used for the other treatments. Additionally, 50 mL of microbial filtrate was added to each treatment to establish a similar microflora (33). Biochar treatments (B, PB, and PBA) received a uniform amendment of 3% biochar (wt/wt). Four germinated maize seeds were sown in each pot, and 10 days after emergence, the seedlings were thinned to two uniform seedlings per pot. The greenhouse maintained a temperature of 28°C/15°C day/night. Each pot was daily watered with distilled water to maintain soil moisture at approximately 60% of field capacity. The positions of all trial pots were randomly rotated every 2 weeks.

After 2 months of growth, measurements were taken for maize height and stem diameter. Shoots and roots were harvested separately and washed carefully with deionized water. One fresh root subsample was collected for the determination of mycorrhizal colonization rate using the modified grid line intersect technique (34). The shoot and root biomass was determined after oven drying at 80°C for 72 hours. Oven-dried plant samples (shoots and roots) of 0.5 g were milled and digested in a concentrated HNO_3_/HClO_4_ (4:1, vol/vol) mixture for 3 hours. Phosphorous and Pb concentrations in maize roots and shoots were quantified using inductively coupled plasma-atomic emission spectrometry (ICP-AES).

The soil from each pot was homogenized and finely powdered through a 100-mesh sieve. Soil pH was measured using distilled water and a pH meter (1:2.5 wt/vol). The CEC was determined using the sodium acetate/ammonium method (35). Soil-available phosphorus was assessed using the molybdenum blue method (36). Soil-available N (NH_4_^+^–N and NO_3_^−^–N) was measured using the dual-wavelength spectrophotometric method and the indophenol blue method (37). Soil dissolved organic carbon (DOC) was determined using a multi N/C 2100/2100S TOC Analyzer. The microbial biomass carbon (MBC) and microbial biomass nitrogen (MBN) were measured using the chloroform fumigation-extraction method (38). Soil diethylenetriaminepentaacetic acid (DTPA) and CaCl_2_-extractable Pb were measured using ICP-AES. The Pb fractions in soil were extracted using the European Community Bureau of Reference sequential extraction method (39), which included four different Pb forms: acid-soluble, reducible, oxidizable, and residual.

The influence of biochar and AMF on the plant’s ability to take up and translocate Pb was evaluated based on the bioconcentration factor and translocation factor, respectively (40). They were calculated as follows:


(1) $$BCF=C_{shoot}/C_{soil}$$

(2) $$TF=C_{shoot}/C_{root}$$

where *C*_shoot_, *C*_root_, and *C*_soil_ are the concentrations of Pb in maize shoots, roots, and soil, respectively.

The Pb immobilization index (41), representing the percentage of reduction in extracted Pb concentration in the soil, was calculated as follows:


(3) Pb−IMMi (%)=Extracted Pb in (control−sample)/Extracted Pb in control×100

### Statistics

All data are presented as mean ± standard errors (*n* = 5). Prior to analysis, all data were checked for normality and homogeneity of variance and transformed when necessary. Data analysis was conducted using one-way analysis of variance via SPSS 25.0 statistical software, and treatment means were compared using Duncan’s test at a significance level of *P* < 0.05. The figures were created using SigmaPlot 12.5.

## RESULTS

### Mycorrhizal colonization, maize growth, and phosphorous uptake

**Fig 1:**
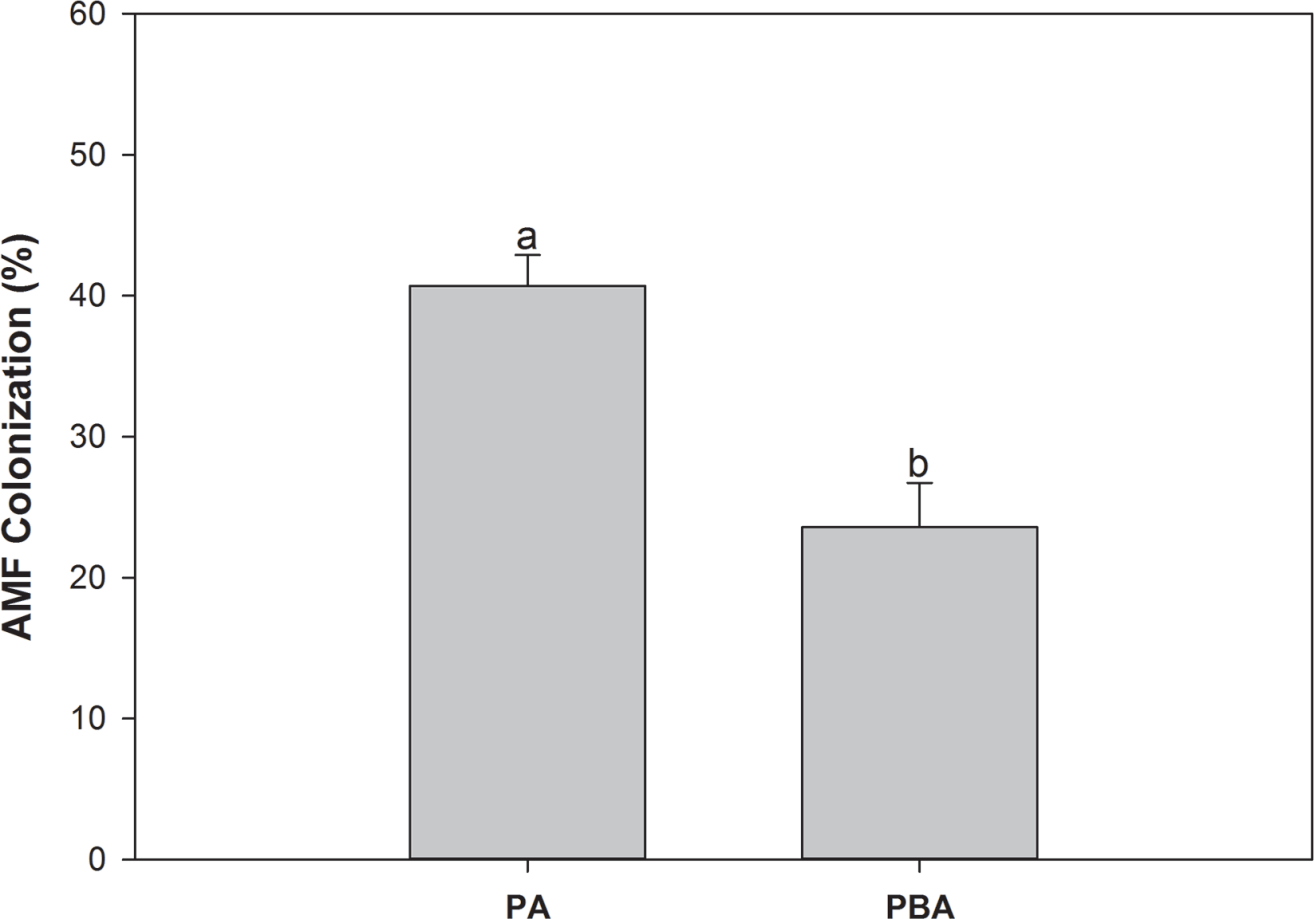
The effect of biochar on mycorrhizal colonization rate. Different letters indicate significant differences between treatments by Duncan's test (*P* < 0.05).

In the treatments without inoculation, there was no observable AMF colonization in the maize roots. The mycorrhizal colonization rate in the AMF-inoculated maize ranged from 18.00% to 45.06% (Fig. 1). Biochar addition considerably reduced mycorrhizal colonization rate by 42.09%. Additionally, a robust negative correlation existed between root mycorrhizal colonization rate and the soil’s available phosphorus content, as depicted in Fig. S1.

**Fig 2:**
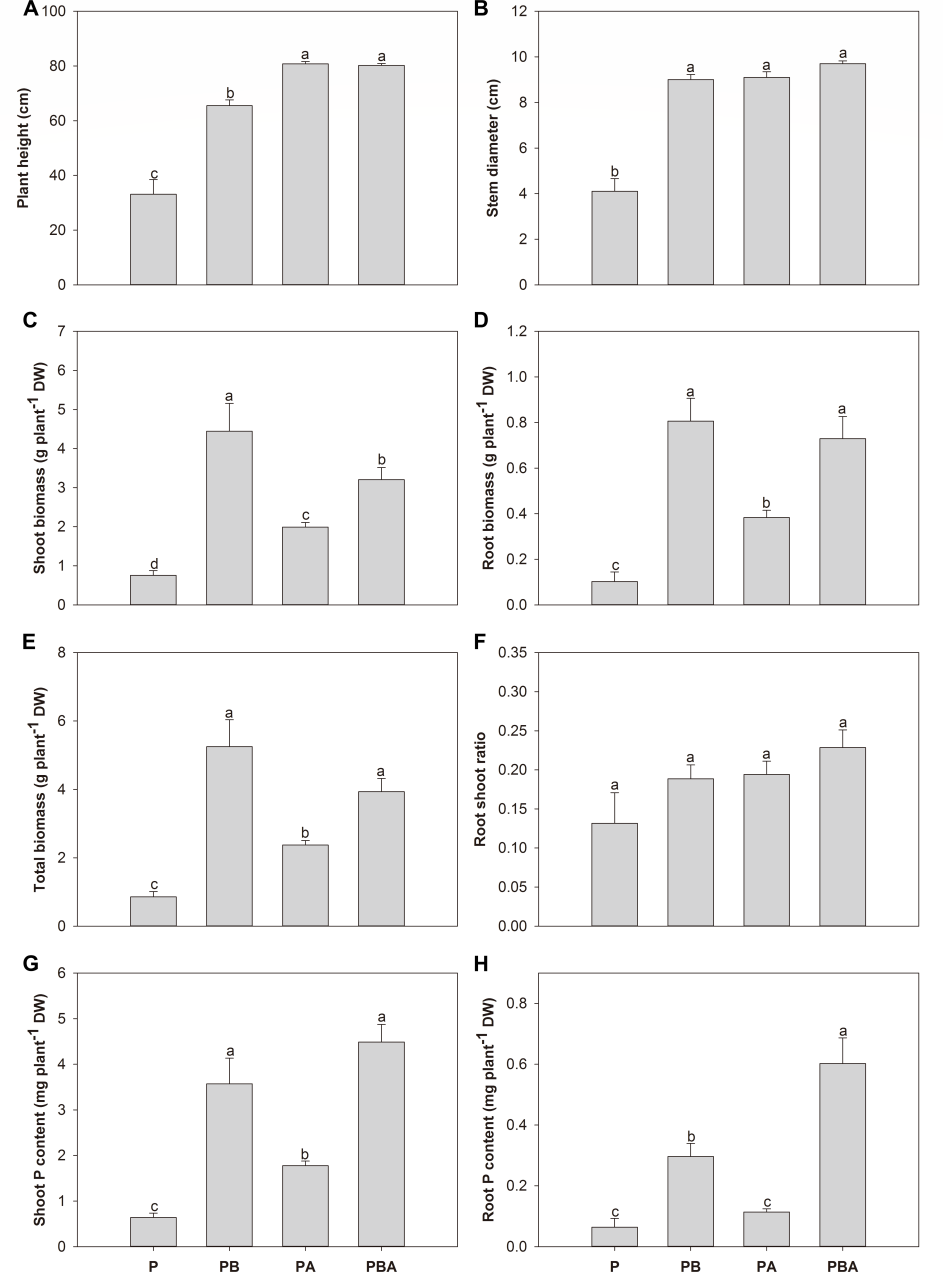
The effects of biochar and AMF on plant height (**A**), stem diameter (**B**), shoot biomass (**C**), root biomass (**D**), total biomass (**E**), root shoot ratio (**F**), phosphorous content in maize shoot (**G**), and root (**H**). Different letters indicate significant differences among treatments by Duncan's test (*P* < 0.05). DW, dry weight.

Biochar, AMF, and their combined treatment significantly increased maize height by 97.89%, 144.11%, and 142.30%, respectively, when compared to plant cultivation alone (Fig. 2A). Maize stem diameter was considerably increased, by a factor of 1.20, 1.22, and 1.37, following the application of biochar, AMF, or both, respectively (Fig. 2B). Single application of biochar, AMF, and their combined application resulted in a factor of 4.84, 1.62, and 3.22 increase in maize shoot biomass, respectively (Fig. 2C). Maize shoot biomass was the highest under biochar treatment, followed by the combined application of biochar and AMF, then AMF treatment, and finally, plant cultivation only (Fig. 2C). In terms of maize roots and overall biomass, similar patterns were also observed (Fig. 2D and E). No significant difference was observed in the root shoot rate among all treatments (Fig. 2F). With biochar, AMF, and combined treatment, the maize shoot phosphorous content was considerably raised by a factor of 4.60, 1.78, and 6.03, respectively (Fig. 2G). Biochar alone and combined application of biochar and AMF significantly increased the maize root phosphorous content by a factor of 3.52 and 8.09, respectively (Fig. 2H).

### Soil features

**Fig 3:**
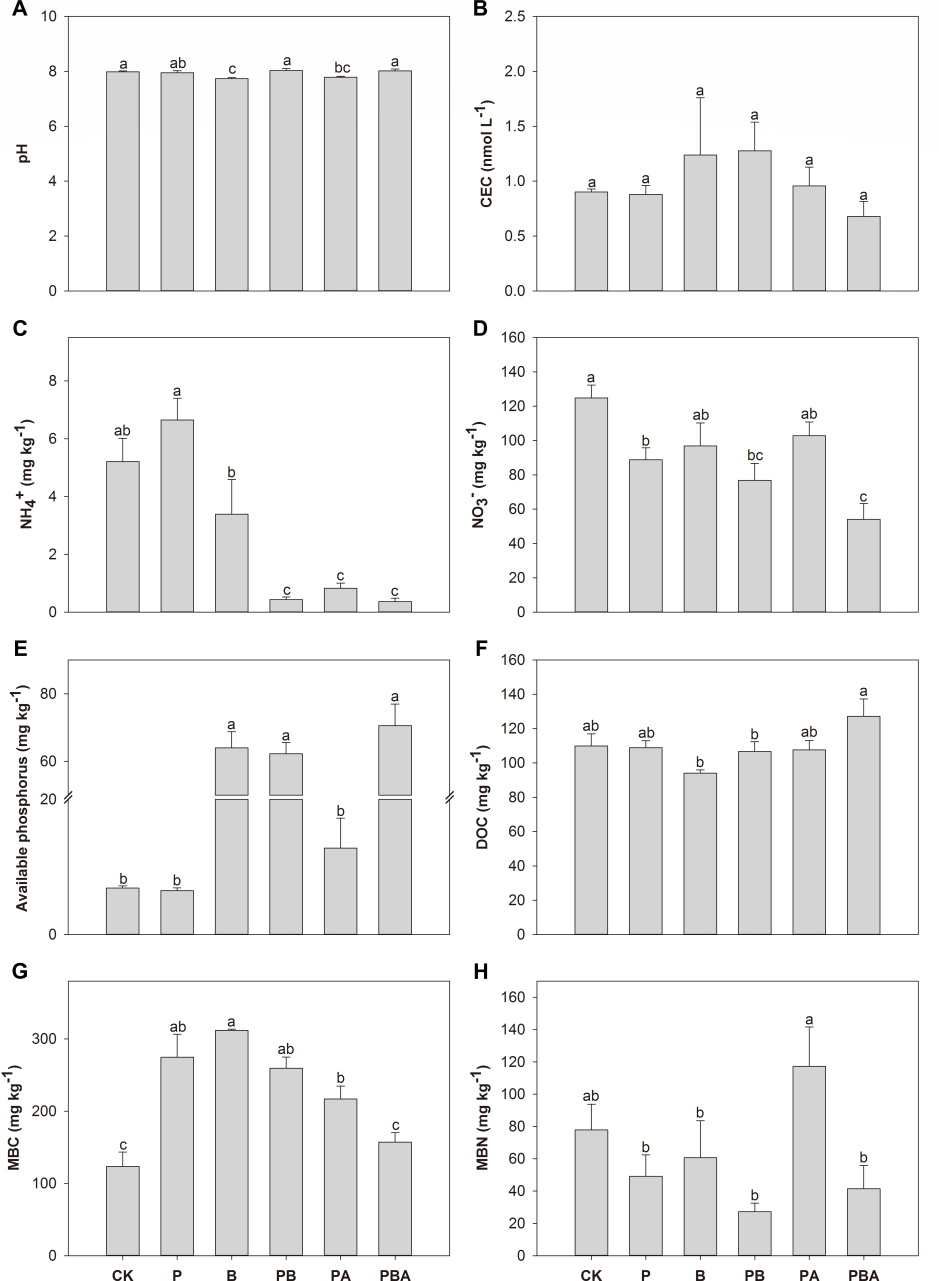
The effects of plant cultivation, biochar, and AMF on soil pH (**A**), CEC (**B**), NH_4_^+^ (**C**), NO_3_^−^ (**D**), available phosphorus (**E**), DOC (**F**), MBC (**G**), and MBN (**H**). Different letters indicate significant differences among treatments by Duncan’s test (*P* < 0.05).

Soil pH, CEC, DOC, and MBN levels remained largely unchanged across all treatments, as illustrated in Fig. 3A, B, F, and H. When compared to the control, soil NH_4_^+^ and NO_3_^−^ levels exhibited significant reductions following plant cultivation with biochar, as well as the combined application of biochar and AMF, as shown in Fig. 3C and D. Furthermore, in the presence of AMF inoculation during plant cultivation, soil NH_4_^+^ exhibited a substantial reduction of 84.07%, whereas NO_3_^−^ decreased by 28.83% during plant cultivation. Soil-available phosphorus was significantly increased by biochar addition, plant cultivation with biochar, and plant cultivation with biochar and AMF (Fig. 3E). Soil MBC exhibited significant increases in response to all treatments except in cases of plant cultivation with the concurrent presence of biochar and AMF (Fig. 3G).

### Soil Pb fractions and bioavailability

**Fig 4:**
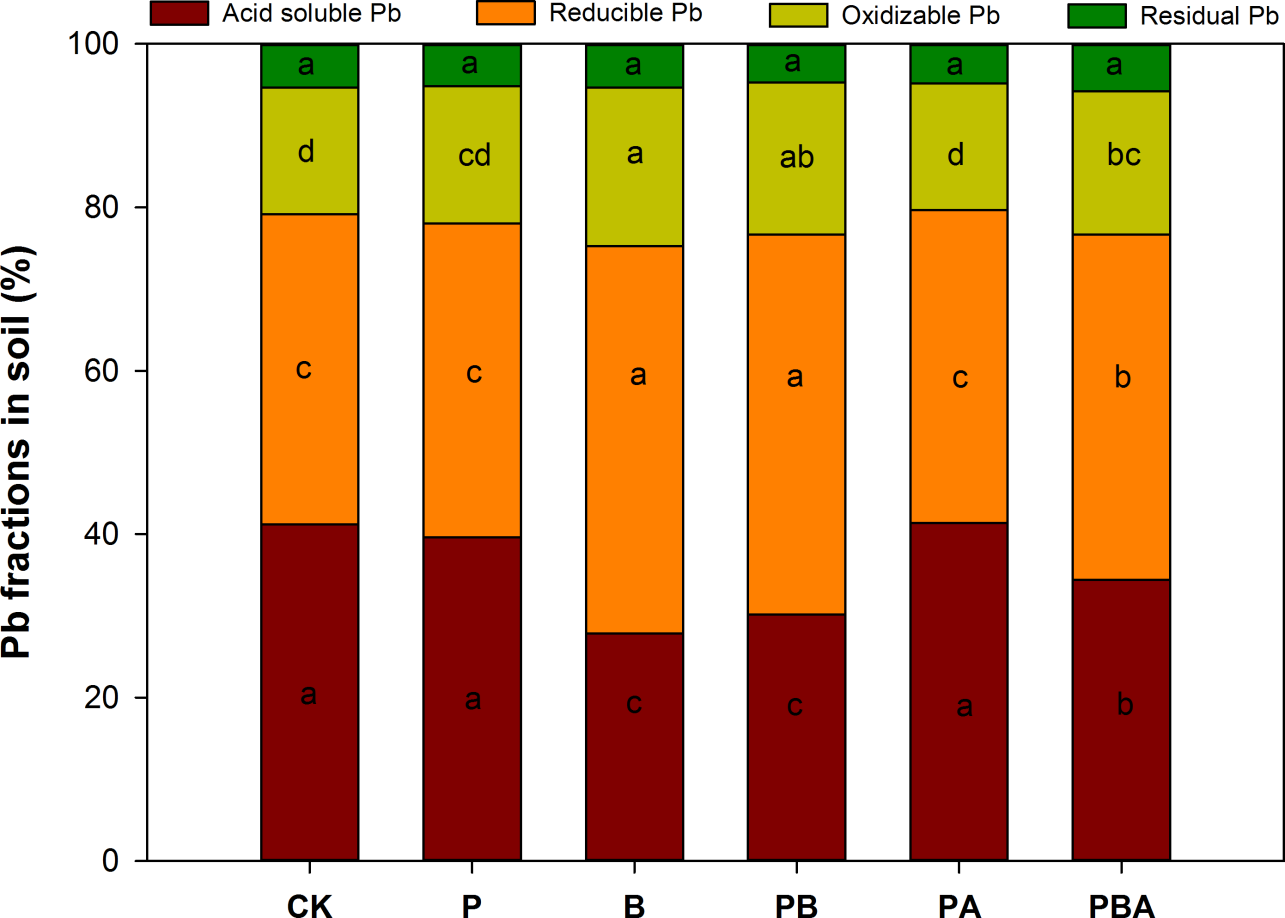
The effects of plant cultivation, biochar, and AMF on soil Pb fractions. Different letters indicate significant differences among treatments by Duncan’s test (*P* < 0.05).

Figure 4 presents the results of sequential soil Pb extractions under various treatments. The analysis revealed that the majority of soil Pb existed in the acid-soluble (26.71% to 43.09%) and reducible (36.43% to 48.37%) fractions, respectively, as contributors to the total Pb concentration. Relative to control, neither plant cultivation nor the combination of plant cultivation with AMF significantly altered the Pb fractions. Conversely, treatments incorporating biochar consistently exhibited a reduction in the proportion of acid-soluble Pb, while concurrently experiencing significant increases in the reducible and oxidizable fractions, with the reducible fraction being the most pronounced. The quantity of residual Pb bound in the soil remained consistent across all treatments.

**Fig 5:**
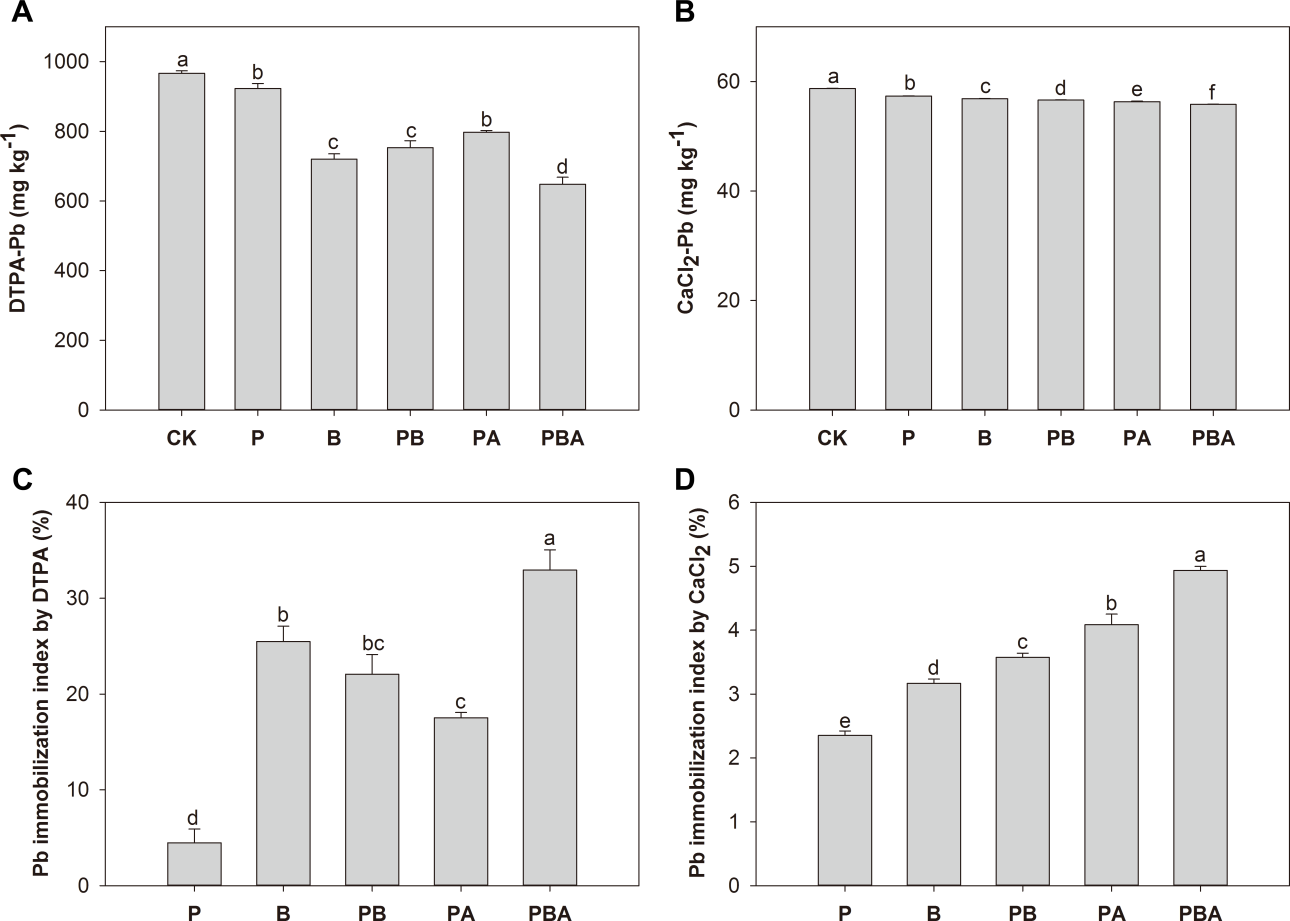
Soil DTPA-extractable (**A**) and CaCl_2_-extractable Pb concentration (**B**) and immobilization index indicated by DTPA (**C**) and CaCl_2_ (**D**) under different treatments. Different letters indicate significant differences by Duncan’s test among treatments (*P* < 0.05).

Bioavailable Pb concentrations were assessed using the DTPA and CaCl_2_ extraction methods. In all cases, there was a notable decrease in soil DTPA-Pb concentration across treatments, with a Pb immobilization index of 4.48% for plant cultivation, 25.49% for biochar addition, 22.07% for plant cultivation with biochar, 17.52% for plant cultivation with AMF, and 32.94% for plant cultivation with biochar and AMF (Fig. 5A and C). Furthermore, when compared to plant cultivation alone, plants inoculated with AMF, biochar, and their combination consistently exhibited notably lower DTPA-Pb concentrations, with the lowest concentration observed in the treatment involving both biochar and AMF. Like DTPA-Pb, all treatments reduced the soil CaCl_2_-Pb concentration (Fig. 5B). Compared with control treatment, soil CaCl_2_-Pb levels were reduced by 2.35% in plant cultivation, 3.24% in the presence of biochar, 3.69% in plant cultivation with biochar, 4.24% in plant cultivation with AMF, and 5.15% in plant cultivation with both biochar and AMF (Fig. 5B and D). Notably, the lowest CaCl_2_-Pb levels in the soil were observed in the treatment involving both biochar and AMF following plant cultivation, as indicated in Fig. 5B and D.

### Pb uptake and transfer

**Fig 6:**
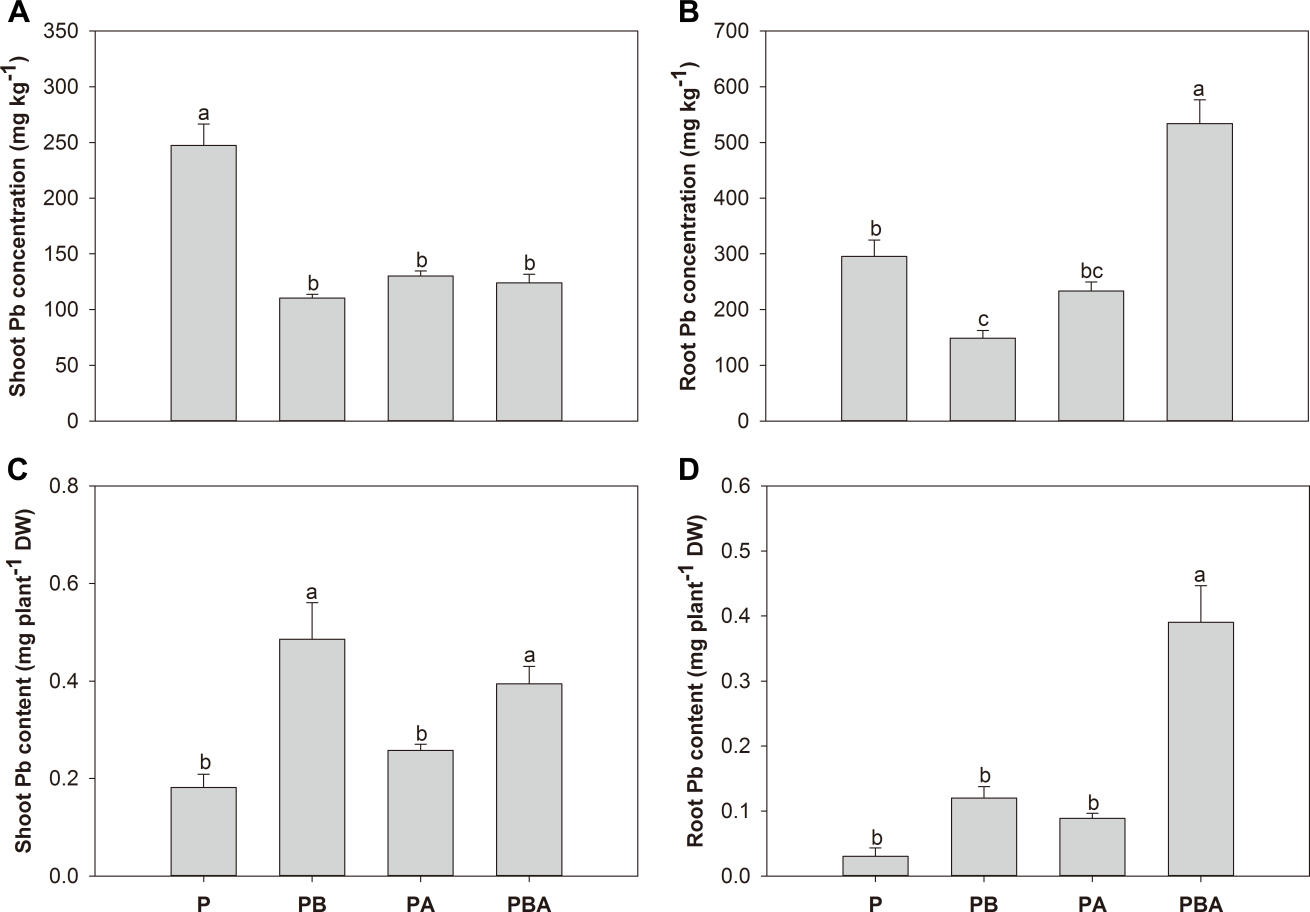
The effects of biochar and AMF on maize shoot Pb concentration (**A**), root Pb concentration (**B**), shoot Pb content (**C**), and root Pb content (**D**). Different letters indicate significant differences among treatments by Duncan’s test (*P* < 0.05).

In comparison to plant cultivation alone, the addition of biochar, AMF, and their combined treatment resulted in significant reductions in maize shoot Pb concentration by 55.41%, 47.31%, and 49.86%, respectively (Fig. 6A). The introduction of biochar significantly reduced maize root Pb concentration by 49.53%, while the combined application of biochar and AMF substantially increased it by 80.87% (Fig. 6B). In contrast to plant cultivation alone, the maize shoot Pb content was notably higher in the biochar treatment and the combined biochar and AMF treatment (Fig. 6C). The simultaneous application of biochar and AMF substantially increased the maize root Pb content by a factor of 12.00 (Fig. 6D).

**Fig 7:**
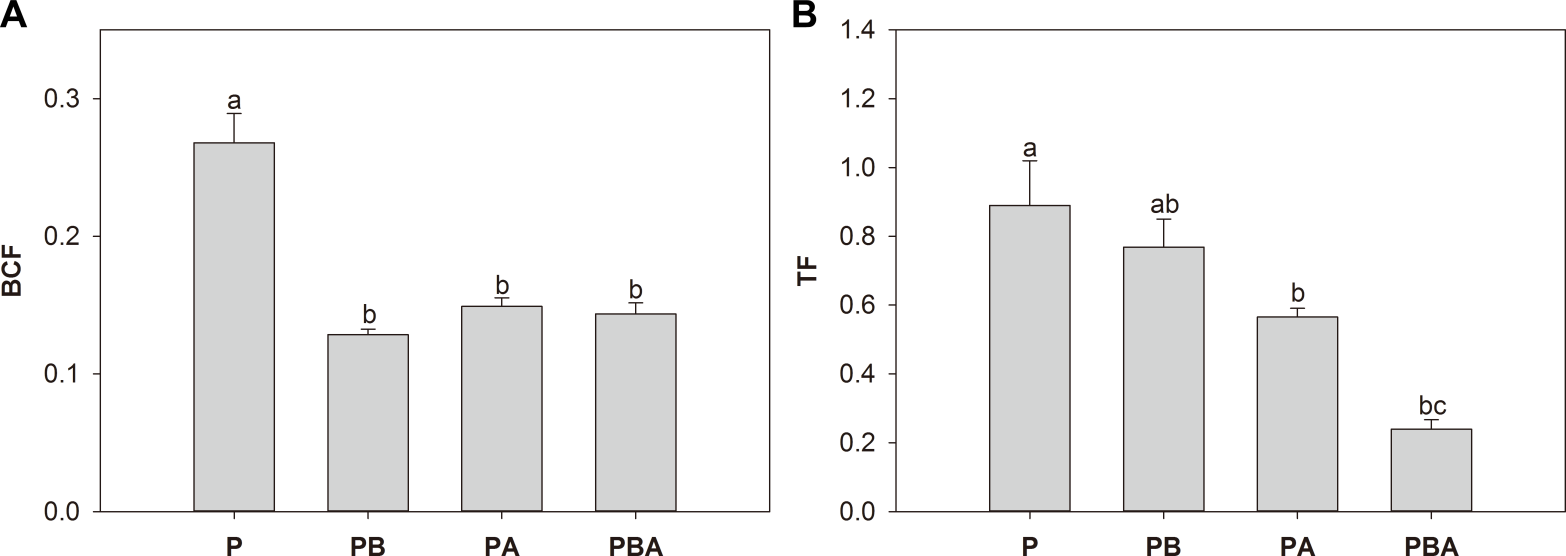
The effects of biochar and AMF on bioconcentration factor (BCF) (**A**) and translocation factors (TF) (**B**). Different letters indicate significant differences among treatments by Duncan’s test (*P* < 0.05).

Bioconcentration and translocation factors of Pb in maize ranged between 0.12 and 0.33 and 0.17 and 1.17, respectively, in all treatments (Fig. 7A and B). In comparison to plant cultivation alone, the bioconcentration factor was notably reduced by 51.85%, 44.44%, and 48.15% with individual application of biochar or AMF and combined application of biochar and AMF, respectively (Fig. 7A). In contrast, AMF inoculation and the combined application of biochar and AMF led to substantial reductions in the translocation factor by 35.96% and 73.03%, respectively (Fig. 7B).

## DISCUSSION

In this study, a decrease in root fungal colonization was evident in the presence of biochar. The decrease in mycorrhizal colonization may be attributed to the increased availability of phosphorous introduced by the addition of biochar. Biochar inherently harbors a quantifiable amount of phosphorus, and its introduction into the soil contributes to an augmentation in phosphorus availability. Simultaneously, the application of biochar elicits a rise in soil pH, thereby amplifying the solubility of phosphate and, consequently, augmenting the overall availability of phosphorus (42, 43). The higher levels of available phosphorous in the soil are detrimental to mycorrhizal infection (44). This is further supported by the negative association between mycorrhizal root colonization and soil-available phosphorous content (Fig. S1).

Maize plants inoculated with AMF exhibited greater plant height, stem diameter, and biomass compared to non-inoculated plants. The mechanism may be related to the ability of the AMF extraradical hyphal network to increase host plants’ water and nutrient uptake (i.e., increased maize shoot phosphorous content). AMF inoculation may result in maize obtaining more nutrients, enhancing its resilience to Pb stress. Moreover, soil enriched with biochar demonstrated significantly enhanced plant growth in the presence of Pb stress. In line with the current findings, earlier research demonstrated that adding biochar to Pb-contaminated soils promoted plant growth and yield in a variety of species (45). Previous research reported that biochar addition can increase aeration conditions, soil fertility, water-holding capacity, microbiological activity, and aggregate stability (14). Since biochar is a substantial source of phosphorous in this study (Table S1), its addition to the soil augmented the availability of phosphorous, resulting in a notable increase in phosphorous uptake by maize roots and shoots. Additionally, numerous studies have demonstrated that biochar enhances soil microbial activity, which can influence both soil quality and plant growth (46). As demonstrated in this study, biochar addition significantly improved the amount of soil microbial carbon.

It is widely recognized that the overall physiological and toxic effects of heavy metals on biological systems primarily hinge on the speciation and availability of HMs, rather than their total content (47). In this study, soil after plant cultivation exhibited significantly lower Pb bioavailability (DTPA-Pb and CaCl_2_-Pb) than the control treatment. A previous study showed that the oxalate compounds produced from *Oryza sativa* rhizosphere decreased the Pb bioavailability, which may constitute an essential Pb tolerance mechanism in rice (48). The maize may secrete polysaccharide-based viscous substances, which adsorb and precipitate lead ions outside the roots, thereby reducing lead mobility in soil and its availability to plants (49). A similar complex formation between citrate exudation and Cd was also observed in maize roots, resulting in reduced Cd bioavailability (50).

The present study demonstrated a significant decrease in soil-bioavailable Pb due to AMF inoculation in comparison to treatments without AMF. This is in line with other research, which showed the efficiency of AMF in reducing Pb bioavailability and toxicity (24). AMF was reported to assist host plants in resisting Pb toxicity by promoting the release of organic small molecules that interact with Pb (e.g., metallothioneins, glutathione, and phytochelatins) (51). On the other hand, AMF spores and extraradical mycelia can help to bind Pb by forming surface complexation with Pb (52).

In the current investigation, the addition of biochar had no discernible impact on residual Pb. Nevertheless, the addition of biochar led to a transformation of Pb forms from the acid-soluble fraction to less reactive forms (reducible and oxidizable fractions), regardless of plant cultivation and AMF inoculation. The HM acid-soluble fraction is regarded to be the most mobile portion because it is quickly released into the aquatic environment and changed into free ions (53). Therefore, the changes in Pb fractions by biochar addition can reduce the potential risk of Pb to the environment. Similarly, the transformation of Pb from the acid-soluble fraction to other fractions was observed after biochar addition (54). In addition, the findings demonstrated that adding biochar considerably decreased the bioavailable Pb levels relative to untreated soil. Biochar is extremely effective at chelating or adsorbing Pb from polluted soil due to its vast surface area and abundance of functional groups (55). This phenomenon may elucidate the changes in Pb fractions and bioavailability observed in this study. Furthermore, within this study, biochar addition was more effective at reducing the bioavailability of Pb to plants than AMF inoculation, as indicated by the lower bioavailable Pb levels.

Lower root Pb concentration was expected in treatment with combined application of AMF and biochar than AMF or biochar alone. However, we found that the combined use of AMF and biochar resulted in a higher root Pb concentration than other treatments. Similar results were found in the root concentration of Pb and other heavy metals in two previous studies (56, 57), depending on soil property and biochar density. The concentration of Pb in maize shoots, as well as the bioconcentration and translocation factors, exhibited significant reductions when biochar and AMF were applied, either separately or in combination. The reduction in Pb concentration in plants and translocation from soil to roots and shoots is considered an important Pb tolerance mechanism of AMF and biochar, which reduces the danger of Pb moving from the soil to the food chain (52). The same phenomenon was found in other heavy metals (54). The decreased concentration and translocation of Pb might result from Pb immobilization by biochar and AMF fungal structures or alteration of Pb speciations and bioavailability in the rhizosphere. This is further supported by a significant negative correlation between maize shoot Pb concentration and soil Pb bioavailability (Fig. S1). Moreover, AMF and biochar significantly promoted soil-available phosphorus, which might intensify the co-precipitation between Pb and phosphorus (58). Nevertheless, it was assumed that the decreased Pb uptake by maize after AMF and biochar addition can be attributed to obvious dilution effects related to increases in maize growth (59).

Despite the reduction in mycorrhizal colonization due to biochar addition, maize displayed a minimal translocation factor when both AMF and biochar were present. This indicates a synergistic impact of AMF and biochar on reducing Pb concentration in maize plants, partly due to their combined effect on Pb immobilization. Previous researchers demonstrated a double-edged effect of biochar on AMF colonization and function (60). On the one hand, biochar may release phosphorus into soil, which inhibits AMF growth. On the other hand, biochar can stabilize available Pb, reducing its toxicity to AMF. Therefore, the effect of biochar on AMF represents a compromise between benefits and detriments. A recent study demonstrated the synergistic effects of biochar and AMF in reducing Pb concentrations in barley shoots, roots, and grains (61). Similarly, the combination of biochar and AMF leads to a greater reduction in maize Pb and Cd concentrations than a single addition of biochar or AMF (62). The synergistic effects of biochar and AMF can be influenced by various factors, including plant genotype and tolerance, AMF origin and tolerance, plant-AMF compatibility, biochar types, and environmental conditions (57, 63).

### Conclusions

Treating Pb-contaminated soils with maize cultivation, biochar, and AMF reduced the bioavailability of the contaminant. Biochar also transformed Pb fractions in the soil, converting the acid-soluble fraction into less reactive forms (reducible and oxidizable fractions). Biochar, AMF, and their combined application significantly increased maize height, stem diameter, biomass, and phosphorus uptake while reducing shoot Pb concentration. Maize treated with AMF and biochar displayed a minimal Pb translocation factor, demonstrating their synergistic effects on Pb phytostabilization. AMF species may differ in how they affect the phytostabilization of different heavy metals. Future studies should aim to elucidate the impact of different AMF species and biochar in naturally occurring heavy metal-polluted environments. In conclusion, our findings support the recommendation of maize cultivation with biochar and AMF as an effective strategy for Pb phytoremediation.

## SUPPLEMENTAL MATERIAL

Supplemental material spectrum.03427-23-s0001.docx Table S1. Biochar properties. Fig. S1. Pearson correlation analysis.

ASM does not own the copyrights to Supplemental Material that may be linked to, or accessed through, an article. The authors have granted ASM a non-exclusive, world-wide license to publish the Supplemental Material files. Please contact the corresponding author directly for reuse.
