# Supplementary material for: Zea mays cultivation, biochar, and arbuscular mycorrhizal fungal inoculation influenced lead immobilization
Source: Microbiol Spectr. 2024 Feb 23;12(4):e03427-23. doi: 10.1128/spectrum.03427-23 (PMC10986566; doi:10.1128/spectrum.03427-23)
Supplement: Supplemental material — Table S1. Biochar properties. Fig. S1. Pearson correlation analysis. [file spectrum.03427-23-s0001.docx]

Supplementary Material

| **Table S1**. Basic properties of biochar used in the pot experiment. | |
| --- | --- |
| Parameters | Biochar |
| pH | 9.6 |
| Total carbon content | 597.7 g kg^-1^ |
| Total nitrogen content | 13.4 g kg^-1^ |
| Total phosphorus content | 2.47 g kg^-1^ |
| Cation exchange capacity | 17.0 cmol kg^-1^ |
| Total Pb content | 4.54 mg kg^-1^ |


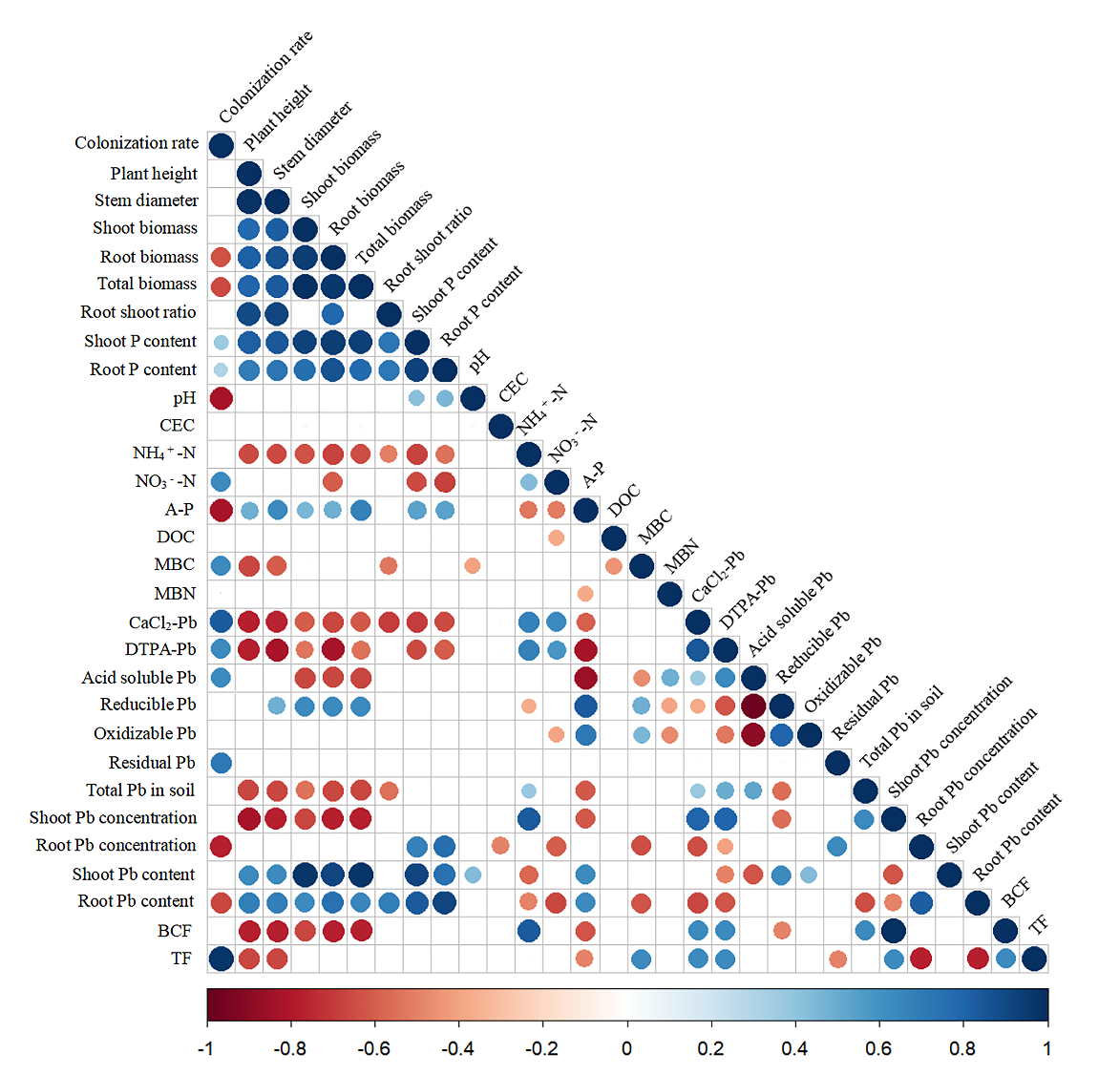


**Fig. S1.** Pearson correlation analysis (*r*) between maize growth parameters and soil properties at harvest. Positive and negative correlations are displayed in blue and red, respectively. Color intensity and the size of the circles are proportional to the correlation coefficients. Null when the correlation is not significant (*p* ≥ 0.05).
